# The Degree of Anxiety and Depression in Patients With Cardiovascular Diseases as Assessed Using a Mobile App: Cross-Sectional Study

**DOI:** 10.2196/48750

**Published:** 2023-10-04

**Authors:** Yongguang Li, Jue Cen, Junxia Wu, Min Tang, Jingyi Guo, Jingyu Hang, Qing Zhao, Gang Zhao, Xiaoli Huang, Beibei Han

**Affiliations:** 1 Department of Cardiology Shanghai Sixth People’s Hospital, Shanghai Jiao Tong University School of Medicine Shanghai China; 2 Center for Hospital Operation Research China Hospital Development Institute Shanghai Jiao Tong University Shanghai China; 3 Shanghai Sixth People’s Hospital Shanghai Jiao Tong University School of Medicine Shanghai China; 4 Department of Comprehensive Statistics Affiliated Nantong Hospital 3 of Nantong University Nantong China; 5 Department of Clinical Research Center Shanghai Sixth People's Hospital, Shanghai Jiao Tong University School of Medicine Shanghai China; 6 Department of Cardiology Shanghai United Family Hospital Shanghai China

**Keywords:** mobile app, anxiety, depression, cardiovascular diseases, Haodf platform

## Abstract

**Background:**

Depression and anxiety are common comorbidities in cardiovascular clinic outpatients. Timely identification and intervention of these mental and psychological disorders can contribute to correct diagnosis, better prognosis, less medical expenses, and improved quality of life. The convenience of online doctor-patient communication platforms has increasingly attracted patients to online consultations. However, online health care and offline health care are very different. Research on how to identify psychological disorders in patients who engage in an online cardiology consultation is lacking.

**Objective:**

This study aimed to explore the feasibility of using a self-rating scale to assess mental illness among patients who consult with a cardiologist online and to compare the differences in anxiety and depression between online and offline patients.

**Methods:**

From June 2022 to July 2022, we conducted follow-up visits with 10,173 patients on the Haodf platform. We conducted detailed consultations with 286 patients who visited the same cardiologist in the outpatient department. We used the self-rated Generalized Anxiety Disorder (GAD-7) and Patient Health Questionnaire (PHQ-9) scales to assess anxiety and depression, respectively. We analyzed the influencing factors related to the degree of coordination of online patients. We also compared the prevalence of anxiety or depression between online and offline patients and analyzed the factors related to anxiety or depression.

**Results:**

Of the 10,173 online consultation patients, only 186 (1.8%) responded effectively. The response rate of the offline consultation patients was 96.5% (276/286). Frequent online communication and watching live video broadcasts were significantly related to effective responses from online patients (*P*<.001). The prevalence of anxiety (70/160, 43.7% vs 69/266, 25.8%; *P*<.001) or depression (78/160, 48.7% vs 74/266, 27.7%; *P*<.001) in online consultation patients was significantly higher than that in offline patients. In bivariate analyses, the factors related to anxiety included female sex, unemployment, no confirmed cardiovascular disease, and the online consultation mode, while smokers and those who underwent COVID-19 quarantine were less likely to present with anxiety. The factors related to depression included female sex, divorced or separated individuals, and the online consultation mode. COVID-19 quarantine was related with a lower likelihood of depression. BMI was negatively correlated with depression. In multiple ordered logistic regression analysis, women were more likely than men to present with anxiety (odds ratio [OR] 2.181, 95% CI 1.365-3.486; *P*=.001). Women (OR 1.664, 95% CI 1.082-2.559; *P*=.02) and online patients (OR 2.631, 95% CI 1.305-5.304; *P*=.007) were more likely to have depression.

**Conclusions:**

Online patients had more anxiety or depression than offline patients. Anxiety was more prevalent in women, the unemployed, and those without confirmed cardiovascular disease. Women and divorced or separated individuals were more prone to depression. Increasing the frequency of doctor-patient communication and participating in video interactions can help improve patient cooperation.

## Introduction

Depression is one of the most widespread health burdens for the general population in China. According to the Global Burden of Disease Study 2019, although the age-standard incidence of depression has declined in China as a whole in the last 3 decades, the incidence of depression among older individuals has increased [1]. Especially for middle-aged and older adults with chronic diseases, the prevalence rates for depression are as high as 36.62% [2]. The incidence of psychosocial disorders such as anxiety and depression in patients with cardiovascular disease (CVD) is significantly higher than that in the general population. A study conducted in a heart failure clinic showed that more than 50% of patients with chronic heart failure had anxiety or depression and more than 10% had severe anxiety or depression [3]. The proportion of depression in patients with heart failure increases with a higher New York Heart Association grade [4]. Depression is common among patients with CVD and is associated with cardiovascular death and disability, leading to higher medical costs and lower quality of life [5]. Therefore, identifying and intervening in psychosocial disorders are important ways to improve the prognosis of patients with CVD.

Among patients who seek medical advice in the outpatient cardiology department due to cardiovascular-related symptoms, some do not have a specific CVD, while others have somatic symptoms of psychological diseases. A study that evaluated the psychological status of patients who visited a chest pain center without a clear cause found that 53.5% of patients had anxiety and 25.4% had depression [6]. Another study showed that 47% of low-risk chest pain patients visiting emergency treatment experience anxiety [7]. These patients cannot improve their symptoms even if structural heart disease is ruled out, resulting in repeated medical visits, a waste of medical resources, and reduced quality of life [7,8]. Therefore, it is the responsibility of cardiologists to identify psychological abnormalities and avoid misdiagnoses and underdiagnoses.

In recent years, with the promotion of the concept of dual heart diagnosis and treatment, an increasing number of cardiologists has begun to attach importance to the identification and treatment of patients’ psychological problems. Through consultation and self-assessment scales, patients’ mental and psychological status can be assessed in doctors’ offices [8]. The development of the internet and the rise of a large number of apps that provide online medical consultation have provided new medical treatment modes for people. After the COVID-19 pandemic, there was a tremendous increase in the online medical community [9]. The Haodf platform is one of the most widely used and influential online medical platforms in China. According to data in the “China Sharing Economy Annual Development Report (2018),” as many as 100 million people use the Haodf platform, with a monthly active number of 1,865,900 cases [10]. Compared with other telemedicine platforms in the world, the Haodf platform is more powerful and convenient to use [11]. Licensed physicians and patients nationwide can freely share this platform. Its concept is similar to offline medical service in China. Patients choose doctors from which they want to receive a consult on the platform. They can select synchronous consultation methods (video, internet phone) or delayed replies (text, voice). Doctors can also promote the popularization of science (text or video) on the platform. Medical services include consultation, health education, diagnosis, prescription of drugs, guidance, and referral. Based on China’s well-established infrastructure, patients and doctors can communicate anytime and anywhere while also protecting personal privacy. However, online consultation is different from offline medicine. Communication between doctors and patients through voice and text is not as convenient, timely, and sufficient as face-to-face consultations. Cardiologists face challenges in identifying and treating mental and psychological disorders among online patients.

In this study, we conducted a questionnaire survey with patients who have sought medical service from 1 registered cardiovascular physician on the Haodf platform within 3 years, and we scored responses on self-rated anxiety and depression scales. The prevalences of anxiety and depression were analyzed and compared with those for patients who were treated by the same cardiovascular physician in the offline office. This study explored the feasibility of using the self-rated scales to identify psychosocial disorders in patients undergoing online consultation.

## Methods

### Participants and Study Design

The STROBE guidelines were followed to conduct a cross-sectional study to evaluate the degree of anxiety and depression in patients with CVD using a mobile app. Methodology projects followed a voluntary design approach. The first step was to explore the clinical characteristics of the online and offline patients. The second step was to explore the correlated factors between anxiety and depression, and this study presents the final step of exploring the influencing factors of the degree of cooperation by online patients. The independent variables assessed were age, sex, BMI, marital status, current smoking status, alcohol intake, occupation, nationality, home quarantine, consultation mode, and CVD. All patients who presented to this physician in the cardiology department were considered for inclusion in the study population. These patients did not have indications for emergency treatment. Patients aged 18 years to 75 years were considered eligible if they were willing to participate in the study and sign an informed consent form. According to whether there was organic CVD, the patients who visited the doctor’s clinic were divided into patients with organic CVD and patients without organic CVD. Some patients have regular follow-up visits, and other patients see the doctor due to discomfort. The exclusion criteria were patients diagnosed with emergent cardiac conditions such as acute myocardial infarction, sick sinus syndrome, pulmonary embolism, aortic coarctation, and severe myocarditis. In addition, patients were excluded if they had participated in other clinical trials within 3 months; had been diagnosed with schizophrenia, obsessive-compulsive disorder, and other psychiatric disorders; or refused to participate in this study. This cross-sectional study used convenience sampling, a nonrandom sampling method. Due to the study design, a double-blind study was not possible. A questionnaire survey and self-assessment scales for psychiatric disorders were distributed to patients who consulted with the same registered cardiologist on the Haodf platform or the same cardiologist in the outpatient cardiology department at Shanghai Sixth People’s Hospital. Patients who sent back valid responses from June 2022 to July 2022 were included in the online or offline treatment group. All participants completed the questionnaire, and collected information included basic information, medical history, concomitant diseases, and medication. Questionnaire surveys, personnel training, and our database management were all strictly controlled in clinical research centers.

### Ethics Approval

The study was conducted in a department of a university hospital. The research was approved by the Ethics Committee of Shanghai Sixth People’s Hospital (approval number: 2022-KY-188(k)). This observational study involving human participants was in accordance with the ethical standards of 1964 Helsinki Declaration and its later amendments or comparable ethical standards. Written informed consent was obtained from all participants prior to inclusion in the study. When the patient signed the informed consent, we also stated that the original informed consent (or the institutional review board) allows secondary analysis without additional consent. The study data were anonymous. There was no research fee in the study, and the participants were not paid.

### Criteria of the Anxiety and Depression Scales

The self-rated Patient Health Questionnaire (PHQ-9) and Generalized Anxiety Disorder (GAD-7) scales were used to assess patients’ depression and anxiety, respectively. A GAD-7 scale score of 0-4 indicates no anxiety, 5-9 indicates mild anxiety, 10-13 indicates moderate anxiety, 14-18 indicates moderate or severe anxiety, and 19-21 indicates severe anxiety. A PHQ-9 scale score of 0-4 indicates no depression, 5-9 indicates mild depression, 10-14 indicates moderate depression, 15-19 indicates moderate and severe depression, and 20-27 indicates severe depression. The cutoff value for the second classification was based on the scale's score to classify anxiety and depression. The cutoff values were all 4, with scores ≤4 indicating no anxiety and depression and scores >4 indicating the presence of anxiety or depression.

### Outcomes

Anxiety and depression measured using the PHQ-9 and GAD-7 and their related factors in online and offline patients with CVD were the main research objectives, and the secondary research objective was to observe the cooperation with the network by online patients and the associated factors.

### Sample Size

To calculate the sample size (n), we used n=[tα^2^*P*(1-*P*)]/d^2^, where d is the allowable error, *P* is the estimation rate, and d=r*P* (r is the allowable error coefficient of *P*), usually set as α=.05 and r=0.1. The literature shows the prevalence of anxiety and depression in heart disease patients is >50%. Therefore, the estimated sample size for this study was *P*=50%, resulting in a sample size of 400. The actual sample size for this study was 426 patients.

### Statistical Analyses

For database management and statistical analysis, we used SPSS (version 22.0; IBM Corp). Departure from normality was tested using the Shapiro-Wilk statistic. Parameters of a normal distribution are described with mean (SD). Parameters of a non-normal distribution are described as the median and quartile. Counted distributions are described as percentage representations. For parameters with a non-normal distribution, the Mann-Whitney *U* test was used to compare between 2 groups. For parameters with a normal distribution, the Student *t* test was used to compare between groups. To compare counted parameters between groups, the chi-square test was used. We performed multiple ordered logistic regression analysis to identify factors related to anxiety and depression, as well as factors related to the patient’s degree of cooperation. The multiple ordered logistic regression analysis used in this study was based on bivariate analysis, and statistically significant variables were included in the regression model. The factors of BMI, marital status, current smoking, occupation, home quarantine, and CVD were corrected, in which the continuous variable BMI was statistically processed as a covariate. A *P* value <.05 was considered statistically significant.

## Results

### Baseline Patient Characteristics

**Figure 1:**
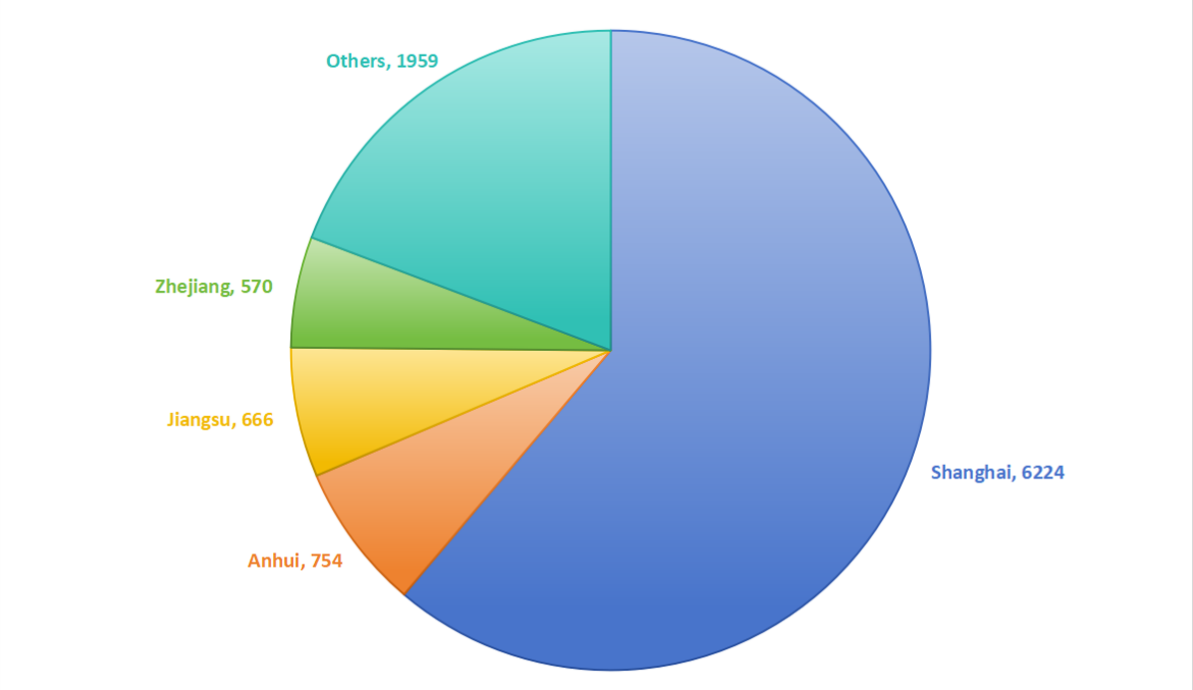
The national distribution of online patients.

**Figure 2:**
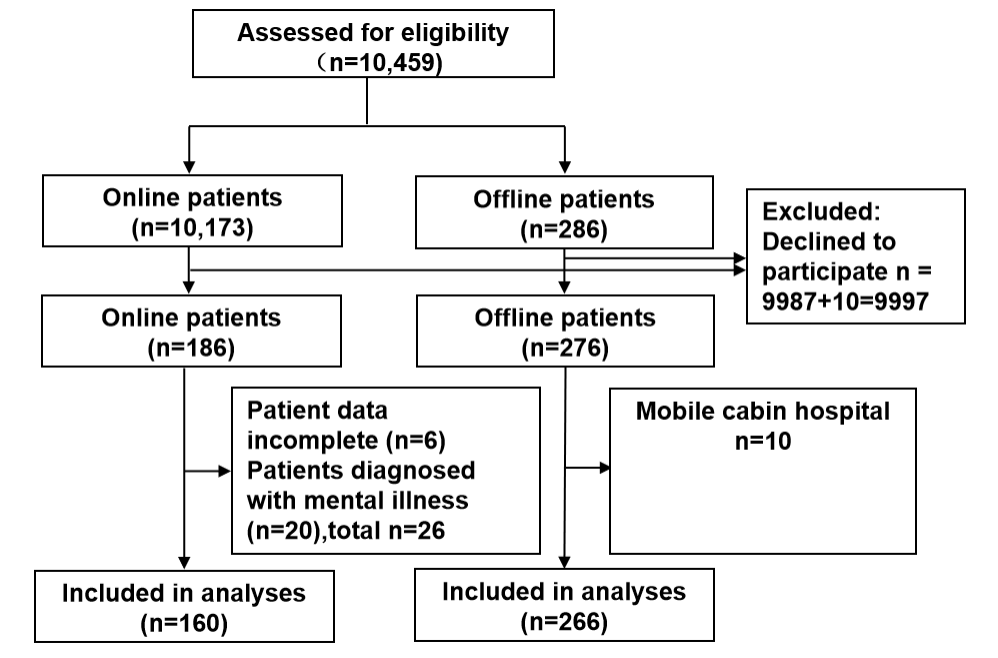
Flow diagram describing the design of the study enrollment and data analysis.

During the past 3 years, a total of 10,173 patients underwent online consultation through the Haodf platform, with a geographical distribution mainly in Shanghai, Anhui, Jiangsu, and Zhejiang, accounting for 80% of the total (Figure 1). However, nearly 20% of the online patients also came from other parts of the country. Among them, 9987 cases did not respond to the questionnaire and were deemed to have refused to participate in the study. The questionnaire was accepted by 186 cases, representing a cooperation rate of 1.8%. A total of 26 cases were excluded, including 6 cases with incomplete questionnaires and 20 cases with confirmed mental or psychiatric diseases. The remaining 160 cases were included in the study and formed an online visit group. A total of 286 patients participated in offline visits, of which 10 refused to participate in the study, representing a cooperation rate of 96.5%. Excluding those who refused to participate in the study and 10 patients who had previously been quarantined in mobile cabins, the remaining 266 cases were included in the analysis and formed an offline treatment group (Figure 2).

There was a significant difference in age between the 2 groups, with younger people involved in online visits. Marital status was also different, with a higher proportion of online patients who were unmarried or divorced. Their occupational composition was significantly different, with a higher proportion of mental health workers and students among those who visited online medical practitioners, while there was a higher proportion of retirees among those who visited offline medical practitioners. The sex ratio and smoking, alcohol consumption, and underlying CVD rates were similar between the 2 groups (Table 1).

**Table 1:** Analysis of clinical characteristics of the 2 groups in the sample.

Characteristic		Sample size (n=426)	Online patients (n=160)	Offline patients (n=266)	Statistic	*P* value
Age (years), median (quartiles)		54.0 (41.0-66.0)	47.5 (38.0-62.8）	58.0 (45.0-68.0)	*z*=–4.161	<.001
BMI (kg/m^2^), median (quartiles)		23.8 (21.3-26.3)	23.1 (20.8-26.1)	24.2 (21.8-26.4)	*z*=–1.969	.049
**Sex, n (%)**					*χ*^2^_1_=1.270	.27
	Male	206 (48.4)	83 (51.9)	123 (46.4)		
	Female	220 (51.6)	77 (48.1)	143 (53.6)		
**Current smoking status, n (%)**					*χ*^2^_1_=0.073	.79
	Yes	64 (15)	25 (15.6)	39 (15)		
	No	362 (85)	135 (84.4)	227 (85)		
**Alcohol abuse, n (%)**					*χ*^2^_1_=0.035	.85
	Yes	36 (84.5)	13 (8.1)	23 (9)		
	No	390 (15.5)	147 (92)	243 (91)		
**Marital status, n (%)**					*χ*^2^_3_=13.121	.003
	Married/cohabitating	359 (84.3)	124 (77.5)	235 (88.4)		
	Unmarried	45 (10.6)	27 (16.9)	18 (6.7)		
	Divorced/separated	6 (1.4)	4 (2.5)	2 (0.8)		
	Widowed	16 (37.6)	5 (3.1)	11 (4.1)		
**Nationality,n (%)**					*χ*^2^_1_=2.232	.14
	Han	416 (97.7)	153 (95.6)	263 (98.9)		
	Others	10 (2.3)	7 (4.4)	3 (1.1)		
**Occupation type,n (%)**					*χ*^2^_4_=32.555	.001
	Mental health	190 (44.6)	92 (57.5)	98 (36.7)		
	Manual	29 (6.8)	16 (10)	13 (5.2)		
	Unemployed	23 (5.4)	10 (6.3)	13 (4.9)		
	Student	4 (0.9)	2 (1.3)	2 (0.8)		
	Retired	180 (42.3)	40 (25)	140 (52.4)		
**GAD-7^a^ score,n (%)**					*z*=–9.998	.001
	0-4	287 (67.4)	90 (56.3)	197 (74.2)		
	5-9	86 (20.2)	48 (30)	38 (14.2)		
	10-13	26 (6.1)	12 (7.5)	14 (5.2)		
	14-18	14 (3.3)	6 (3.8)	8 (3)		
	19-21	13 (3.1)	4 (2.5)	9 (3.4)		
**PHQ-9^b^ score,n (%)**					*z*=10.636	.001
	0-4	274 (64.3)	82 (51.3)	192 (72.3)		
	5-9	98 (23)	49 (30.6)	49 (18.4)		
	10-14	30 (7)	18 (11.3)	12 (4.5)		
	15-19	13 (3.1)	5 (3.1)	8 (3)		
	20-27	11 (2.6)	6 (3.7)	5 (1.9)		
**Home quarantine, n (%)**					*χ*^2^_1_=300.818	.001
	Yes	299 (70.2)	33 (20.6)	266 (100)		
	No	127 (29.8)	127 (79.4)	0		
**Confirmed CVD^c^, n (%)**					*χ*^2^_1_=0.801	.37
	Yes	355 (83.3)	130 (81.3)	225 (84.6)		
	No	71 (16.7)	30 (18.8)	41 (15.4)		

^a^GAD: Generalized Anxiety Disorder.

^b^PHQ: Patient Health Questionnaire.

^c^CVD: cardiovascular disease.

### Prevalence of Anxiety and Depression

Among online patients, the proportion of patients with anxiety was significantly higher than that of offline patients (70/160, 43.7% vs 69/266, 25.8%; *P*<.001), with a significantly higher proportion of patients with suspected anxiety (48/160, 30% vs 38/266, 14.2%; *P*<.001) and a slightly higher proportion of patients with mild anxiety (12/160, 7.5% vs 14/266, 5.2%; *P*<.001). The proportion of patients with moderate and severe anxiety was similarly low in both groups. Among online patients, the proportion of patients with depression was significantly higher than that of offline patients (78/160, 48.7% vs 74/266, 27.7%; *P*<.001), with a significantly higher proportion of patients with suspected depression (49/160, 30.6% vs 49/266, 18.4%; *P*<.001) and a significantly higher proportion of patients with mild depression (18/160, 11.3% vs 12/266, 4.5%; *P*<.001). The proportion of patients with moderate and severe depression was low in both groups.

### Influencing Factors for Anxiety and Depression

Bivariate analysis showed that the following groups were more likely to experience anxiety: women (*P*<.001), unemployed patients (*P*=.03), patients without diagnosed CVD (*P*=.03), and online patients (*P*<.001; Table 2). Smokers (*P*=.03) and those undergoing home quarantine (*P*=.001) were less likely to present with anxiety (Table 2). Bivariate analysis showed that the following groups were more likely to experience depression: women (*P*=.003), divorced or separated patients (*P*=.01), and online patients (*P*<.001). However, those undergoing COVID-19 quarantine were less likely to present with depression (*P*<.001). BMI was negatively correlated with depression (*P*=.004; Table 2).

**Table 2:** Analysis of the factors correlated with anxiety and depression.

Characteristic		Anxiety score			Depression score		
		Median (P25-P75)	Statistic	*P* value	Median (P25-P75)	Statistic	*P* value
Age (years)		2.0 (0-6.0)	r=–0.071	.14	3.0 (0.0-6.0)	r=–0.020	.68
BMI (kg/m^2^)		2.0 (0.0-6.0)	r=–0.068	.16	3.0 (0.0-6.0)	r=–0.137	.004
**Sex**			*z*=4.385	<.001		*z*=2.293	.003
	Male	1.0 (0.0-4.0)			2.0 (0.0-5.0)		
	Female	3.0 (0.0-7.0)			3.5 (0.0-7.0)		
**Mobile app**			*z*=4.385	<.001		*z*=5.488	<.001
	Yes	4.0 (0.0-7.0)			4.0 (2.0-9.0)		
	No	0.5 (0.0-5.0)			2.0 (0.0-5.0)		
**Marital status**			*χ*^2^_3_=4.350	.23		*χ*^2^_3_=10.732	.01
	Married/cohabitating	(0.0-6.0)			3.0 (0.0-6.0)		
	Unmarried	2.0 (0.0-7.0)			4.0 (2.0-8.0)		
	Divorced/separated	6.0 (2.8-7.3)			6.5 (3.8-10.0)		
	Widowed	3.0 (0.3-6.0)			4.5 (0.0-7.5)		
**Current smoking status**			*z*=–2.166	.03		*z*=–1.532	.13
	Yes	0 (0-4.0)			2.0 (0-5.0)		
	No	2.0 (0-6.0)			3.0 (0-7.0)		
**Alcohol intake**			*z*=–1.724	.09		*z*=–1.708	.09
	Yes	0 (0-3.8)			1.5 (0-4.8)		
	No	2.0 (0-6.0)			3.0 (0-6.0)		
**Occupational** **type**			*χ*^2^_4_=11.12	.03		*χ*^2^_4_=7.732	.10
	Mental health	3.0 (0-7.0)			3.0 (0-6.0)		
	Manual	0 (0-5.5)			3.0 (0-9.0)		
	Unemployed	5.0 (1.0-9.0)			6.0 (1.0-14.0)		
	Student	1.0 (0-2.8)			3.5 (2.3-4.8)		
	Retired	1.0 (0-5.0)			2.0 (0-6.0)		
**Nationality**			*z*=–0.301	.76		*z*=–0.300	.76
	Han	2.0 (0-6.0)			3.0 (0-6.0)		
	Others	2.0 (0-4.0)			4.0 (0-5.0)		
**Home quarantine**			*z*=–3.292	.001		*z*=–4.251	<.001
	Yes	1.0 (0-5.0)			2.0 (0-5.0)		
	No	3.0 (0-7.0)			4.0 (2.0-9.0)		
**Cardiovascular disease**			*z*=2.127	.03		*z*=1.312	.19
	Yes	1.0 (0-6.0)			3.0 (0-6.0)		
	No	3.0 (0-7.0)			4.0 (0-8.0)		

Multiple ordered logistic regression analysis showed that sex was the only factor associated with anxiety; anxiety occurred 2.181 times more frequently in women than in men (Table 3). Multiple ordered logistic regression analysis also showed that sex and the online consultation mode were associated with depression; female patients had a 1.664 times higher chance of developing depression than male patients, and online patients had a 2.631 times higher chance of developing depression than offline patients (Table 3).

**Table 3:** Multiple ordered logistic regression analysis of anxiety and depression.

Factors^a^	Anxiety score			Depression score		
	OR^b^	*P* value	95% CI	OR	*P* value	95% CI
Sex^c^	2.181	.001	1.365-3.486	1.664	.02	1.082-2.559
Use of the mobile app^d^	—^e^	—	—	2.631	.007	1.305-5.304

^a^BMI, marital status, current smoking, occupation, home quarantine, and cardiovascular disease were corrected.

^b^OR: odds ratio.

^c^Male=1; female=0, with male as the reference category.

^d^Offline=1; online=0, with offline as the reference category.

^e^Not applicable.

### Analysis of the Influencing Factors for the Degree of Cooperation by Online Patients

Questionnaires were distributed to 10,173 online medical patients. Among them, 186 cases sent back their replies and were included in the high cooperation group. The remaining 9987 cases who did not respond were included in the low cooperation group. Multivariate ordered logistic regression analysis showed that watching the doctor's live video broadcast, watching the live broadcast more often, and communicating with the doctor more often were independent influencing factors for high cooperation. Other factors, including the patient’s sex, age, and residential area, as well as whether they had seen this doctor offline before, had no significant correlation with the degree of their cooperation (Table 4).

**Table 4:** Analysis of the influencing factors for the degree of cooperation by online patients.

Characteristic		With questionnaire response	No questionnaire response	Statistic	*P* value
Age (years), median (P25-P75)		46.5 (36.0-62.3)	47.0 (35.0-61.0)	*z*=–0.130	.90
**Sex, n**				*χ*^2^_1_=3.732	.05
	Male	89	5175		
	Female	93	4816		
**Mobile app, n**				*χ*^2^_1_=0.600	.44
	Offline source	83	3854		
	Online source	99	6137		
**Watched** **live video, n**				*χ*^2^_1_=213.567	<.001
	Yes	67	735		
	No	115	9257		
**Coastal region, n**				*χ*^2^_1_=0.552	.51
	Yes	143	8069		
	No	39	1922		
Frequency of doctor-patient communication		57.5 (30.0-100.5)	14.0 (6.0-32.0)	*z*=13.622	<.001
Frequency of watching the live video		0 (0-1.0)	0 (0-0)	*z*=14.871	<.001

## Discussion

### Principal Findings

This study evaluated the prevalences of anxiety and depression among patients who used the Haodf platform to have an online consultation with a cardiologist and compared these patients with patients who visited conventional clinics. The prevalences of anxiety and depression among online patients were significantly higher than those of offline patients. Factors associated with anxiety include the female sex, unemployment, the absence of confirmed CVD, and the online consultation mode, while smoking and undergoing COVID-19 quarantine were less likely to be associated with anxiety. The factors related to depression included the female sex, divorce or separation, and the online consultation mode, while COVID-19 quarantine was less likely to be associated with depression. BMI was negatively correlated with depression. Female sex was associated with both anxiety and depression, while online patients were more likely to experience depression.

### Comparison With Prior Work

There is a high proportion of patients with psychological disorders among patients in a cardiology department. A study showed that 28.5% of the outpatients in the cardiology department had anxiety and 41.9% had depression [12]. According to the Primary Care Evaluation of Mental Disorders (PRIME-MD) scale, 6% of patients in the outpatient cardiology department have severe depression [13]. The prevalence of anxiety or depression is higher in patients with cardio-related symptoms but without structural heart disease. A study found that 42% of patients with chest pain in the outpatient cardiology department have anxiety and 31% have depression. The anxiety or depression score for patients with a negative exercise test was higher than that for patients with a positive exercise test [14]. Patients with structural heart disease also have a high prevalence of anxiety or depression. Previous studies have shown that 42% of outpatients with coronary heart disease have anxiety and 26% have depression [15]. Among outpatients with heart failure with reduced left ventricular ejection fraction, 18.4% had anxiety, and 28.6% had depression [16]. However, in outpatient cardiology departments, the diagnosis and treatment of psychological diseases are currently insufficient [17].

For patients with CVD such as coronary heart disease or heart failure, coexisting anxiety or depression is very harmful: the higher the anxiety and depression score, the higher the probability of hospitalization and a longer hospital stay [18]. Depression and social isolation were independent risk factors for death in outpatients with heart failure [19]. Anxiety and depression can cause heart failure to worsen and increase the risk of hospitalization and death [20]. In patients with hypertrophic cardiomyopathy, anxiety was associated with sudden cardiac death and heart failure, and the highest risk was in those with both anxiety and depression [21]. For patients without structural diseases, if psychological disorders are not diagnosed in time, the consequences are also very serious. Patients repeatedly seek medical treatment, repeatedly check themselves, and overdose on drugs, resulting in poor quality of life and increased use of medical resources [22,23]. Moreover, some studies have shown that, even if structural heart disease is excluded, it cannot reduce the number of patients seeking medical treatment again, which is not beneficial for an improved prognosis [8,24]. Therefore, it is very important to identify highly prevalent mental disorders in cardiovascular outpatients as early as possible.

The use of self-assessment scales is very helpful for cardiovascular physicians to identify mental diseases, and these scales have been widely used with outpatient and inpatient cardiovascular patients [17,25-27]. The PHQ-9 and GAD-7 scales have been verified and widely used for the diagnosis of depression and anxiety, respectively [28,29].

In recent years, online medical services have continuously developed and become increasingly widely used since the COVID-19 pandemic began in 2020 [10]. The Haodf platform is currently the largest online medical consultation platform in China [30]. The advantages of online consultation are obvious. Patients can consult with doctors anytime and anywhere online, without having to go to a hospital, thereby saving time and expenses [31]. Therefore, a considerable number of patients consult cardiovascular doctors through mobile apps. However, there are many differences between online and offline consultations; some are detrimental to doctor-patient communication [11]. For example, doctor-patient communication is limited to text or voice, and it is not possible to observe the patient’s expressions, movements, and other body language. These hinder doctors from fully understanding the patient’s medical history and mental status. In addition, the lack of face-to-face communication makes it more difficult for patients to trust doctors. These factors make it difficult for cardiovascular doctors to diagnose psychological disorders in online consultation patients. This is a new field and challenge. Doctors lack guidance from clinical research, guidelines, or expert consensus, and there have been no relevant reports of previous studies. Therefore, this study is the first exploratory study to evaluate anxiety and depression in online patients using self-rated scales. We found that self-rated scales can be used to assess anxiety or depression in online patients, but this ability was highly dependent on the patient’s cooperation with the doctor. This study also showed that there were significant differences in social characteristics between online and offline patients, including occupation, education level, and family background. The prevalence of anxiety or depression in online patients was significantly higher than in offline patients. Therefore, it is more necessary for cardiologists to pay attention to online patients’ mental and psychological status.

We found that online patients have a very low degree of cooperation in filling out the self-assessment scale, significantly lower than offline patients. Analysis of factors related to the degree of cooperation found that watching live broadcasts from doctors and frequent communication with the doctor were associated with a high degree of cooperation among online patients. Low compliance is a common problem in online medicine, and similar situations exist in other clinical studies, which pose difficulties for diagnosis and subsequent treatment. A study evaluated the use of online interventions, including email, SMS text, and video conferencing, to improve the mental state of healthy people. However, only 69.9% of the participants who received the intervention responded to the mental state questionnaire again, while the participation rate in video conferencing was lower, with only 18.4% participating more than 7 times in 10 video conferences [32]. Some clinical studies using mobile apps for psychological intervention have found that patients’ participation is lower than with an offline intervention [33,34]. Previous research has shown that emotional support value and self-health management value were the 2 most important factors that affect patient participation and loyalty to mobile medical services [35]. Our research showed that patients who have a high degree of cooperation with doctors often watch doctors’ videos and have more frequent communication with doctors. These behaviors, on the one hand, improve patients’ understanding and trust of the doctor, while, on the other hand, they also improved their understanding of diseases. Therefore, they were more cooperative with doctors’ questionnaires. The analysis results of factors related to the degree of cooperation provide us with important hints. First, for patients seeking an online consultation, videos are more attractive. Video science popularization education can achieve better results. Carrying out video consultation services will be more popular. Second, sufficient doctor-patient communication is crucial for both online and offline medical services. It not only helps with correct diagnosis and treatment but also increases trust between doctors and patients. Real-time video communication may be the direction for developing online medical services in the future.

Many studies have confirmed that cardiovascular patients have a higher incidence of psychosocial diseases than the general population, and cardiovascular patients with mental disorders have a worse prognosis. Psychomental diseases and CVD share common factors, including lifestyle factors, autonomic dysfunction, neuroendocrine imbalance, inflammation, insulin resistance, and increased platelet reactivity [5]. Patients with CVD often face complex social and psychological stress, such as lack of social support, trauma, and decline in ability to carry out activities of daily living. These biological, psychological, and social factors can all lead to depression [36]. In recent years, research on the pathophysiological mechanisms of depression has found that oxidative stress and inflammatory activation play a key role in the occurrence of depression [37,38]. These mechanisms are also responsible for the pathophysiological mechanisms of CVD. Therefore, psychosocial diseases and CVD often form a mutually causal and vicious circle. Early identification and optimal management of depression and anxiety are essential to improve the prognosis of patients with CVD.

### Limitations

The limitations of this study are as follows. First, the psychological evaluation of online patients in this study was conducted after the end of the consultation service. It is not clear whether it would be more effective to conduct the psychological evaluation during the online consultation. Second, online patients have a low degree of cooperation. Only 1.8% of patients effectively completed the evaluation scale, which may affect the accuracy of the online patient evaluation.

### Conclusions

This study shows that patients who seek medical consultation with a cardiologist through a mobile app have more anxiety or depression than those who visit outpatient cardiology clinics. Female sex was associated with anxiety or depression, and online patients were more likely to have comorbid depression. Self-assessment scales can help cardiologists identify psychological disorders present in online patients and are beneficial for correct diagnosis and treatment and for improved prognosis. Online patients have a low degree of cooperation with doctors. We can improve patients’ understanding of diseases and trust in doctors by means of video science popularization and increasing the frequency of communication. Changing patients’ understanding of mental and psychological disorders is the key to improving compliance and cooperation. This study suggests that video science popularization is more popular. Therefore, doctors can carry out science popularization with the help of video apps such as TikTok, which can not only enhance their own influence but also, more importantly, more effectively transmit correct disease knowledge and improve the prognosis of patients.

## Abbreviations

CVD: cardiovascular disease
GAD: Generalized Anxiety Disorder
PHQ: Patient Health Questionnaire
PRIME-MD: Primary Care Evaluation of Mental Disorders

## Data Availability

The data sets generated during and/or analyzed during this study are available from the corresponding author on reasonable request.
